# Basics of Functional Echocardiography in Children and Neonates

**DOI:** 10.3389/fped.2017.00235

**Published:** 2017-12-01

**Authors:** Cécile Tissot, Vincent Muehlethaler, Nicole Sekarski

**Affiliations:** ^1^Centre de Pediatrie, Clinique des Grangettes, Chêne-Bougeries, Switzerland; ^2^Service de Pediatrie, Hôpital du Jura, Site de Delémont, Delémont, Switzerland; ^3^Pediatric Cardiology Unit, Department of Pediatrics, Centre Hospitalier Universitaire Vaudois (CHUV), Lausanne, Switzerland

**Keywords:** echocardiography, target, point-of-care, intensive care, neonatology, pediatric, functional, bedside cardiac ultrasound

## Abstract

Functional echocardiography has become an invaluable tool in the pediatric and neonatal intensive care unit. “Point-of-care,” “target,” or “focus” echocardiography allows bedside cardiac ultrasound evaluation of the hemodynamic status of the patient, helps in directing treatment, thus improves patients care. In order to be able to perform functional echocardiography, it is essential to understand the principles of ultrasound, to know the echocardiographic equipment and settings necessary to acquire the images. This article focuses therefore on the basics of cardiac ultrasound. It is meant to give an overview of two-dimensional echocardiographic views, M-mode imaging and Doppler echocardiography for neonatologists and pediatric intensivists. It is richly illustrated for better understanding with some examples of clinical applications of functional echocardiography in the intensive care setting.

## Introduction

Functional echocardiography has become an invaluable tool in the pediatric and neonatal intensive care unit (1–6). Coupled with a clinical examination and monitoring techniques, echocardiography can provide real-time rapid and reliable diagnostic answers that are invaluable to patient care. This non-invasive bedside test has been developed over the past years to practice “point-of-care,” “target,” or “focus” echocardiography and can be used at the patient’s bedside to evaluate cardiac anatomy, to estimate intracardiac pressures and pressure gradients across valves and vessels, to determine the direction of blood flow, pressure gradient across a defect, and to estimate filling pressure and fluid responsiveness. Functional echocardiography is useful to quantify ventricular systolic and diastolic function, to evaluate hemodynamics, to detect the presence of vegetation from endocarditis, to examine for the presence of pericardial fluid and for chamber or vessel thrombi. The characteristics of functional or “point-of-care” echocardiography are summarized in Table 1. The primary barrier to future universal adoption of this operator dependent “stethoscope of the future” is the lack of widespread, efficient, and affordable training solution. The need and demand for training in functional echocardiography has grown in parallel to the expanded use of ultrasound technology and to the development of portable machines. However, as with all tools, a thorough understanding of its uses and limitations are necessary prior to relying on the information it provides.

**Table 1:** Characteristics of point-of-care echocardiography.

Well-defined purpose linked to improving care of patients
Focused and goal-directed
Findings are easily recognizable and easily learned
Quickly performed
Performed at the patient’s bedside

## Principles of Echocardiography

Echocardiography utilizes ultrasound technology to obtain images of the heart and vascular structures. Ultrasounds are sound frequencies higher than the audible range of 20,000 cycles per second. The ultrasound machine consists of a central processor and a transducer which can convert mechanical energy (sound) from electrical energy and *vice versa* through piezoelectric crystals within the transducer. This will generate mechanical energy through a series of sinusoidal cycles of alternating compression and rarefaction. This produced energy passes as a beam to the heart. The beam travels in a direct line until it finds structures with different acoustical impedance, such as between blood and tissue. When this happens, some energy is reflected back to the same piezoelectric crystals and the remaining milder signal is transmitted distally. The reflected energy also called ultrasound echoes will construct the cardiac image. Depending on the tissue characteristics, the portion of acoustic energy transmitted versus reflected will vary. Anything preventing the acoustic signal reflection, for instance air, bone, bandages, or other foreign bodies, will decrease the quality of the images. This is unfortunately a frequent problem in the intensive care unit (7).

## Echocardiography Equipment and Settings

In order to perform functional echocardiography, it is necessary to have an ultrasound machine including a two-dimensional (2D) mode, M-mode, pulsed-wave (PW), and continuous-wave (CW) Doppler as well as color flow Doppler mapping. To be able to evaluate systole and diastole, electrocardiogram gating is required. A storage system and the possibility to measure structures and velocities off-line are advantageous as it permits comparison between the studies.

The motion of the heart requires the use of greater frame rate, which is enhanced by beam focusing. Because of very different patient sizes in pediatrics, ranging from the newborn to the adult patient, several transducers with different frequencies are necessary, ideally from 12 to 2.5 MHz. This allows for imaging at different depths. Higher frequency transducers have higher resolution but less depth of penetration than lower-frequency transducers. High frequency probes focus at depth of 4–5 cm compared to low frequency probes able to focus at 12–16 cm. For this reason, high frequency probes are used in neonates and small children whereas low frequency probes are used in adults with mid-range frequency transducer used in toddlers or small children.

Special care should be taken to adjust the ultrasound machine to optimize the echo image. Most echocardiography machines have a single button to adjust and optimize the image. There remain some simple adjustments that can help optimize image acquisition. The choice of transducer is the most important for enhancing image quality. It is important to choose appropriate presets, particularly for color Doppler. The image depth should be adjusted so that the heart fills the viewing screen. The 2D gain is used to adjust the strength of the returning echoes and may be controlled in two ways. Overall gain may be changed to enhance the brightness of the image. Additionally, time-gain compensation allows changes to the gain at various depth of interest and is controlled by a set of horizontal slide bars. The contrast can be improved by adjusting the compression or dynamic range. Echo machines also allow adjustment to the beam focus.

## The Anatomical Echocardiographic Examination of a Normal Heart

In order to remove respiratory artifacts, patients are placed in a left lateral decubitus position whenever possible. All planes refer to the axis of heart and not to its position within the body. The different views are obtained from standard windows. Segmental approach is the preferred way of imaging the heart, especially for checking cardiac anatomy, and the situs and laterality need first to be determined, especially in the newborn. Thereafter, each cardiac structure is examined and described from the systemic and pulmonary venous return through the atria, ventricles, and great vessels.

Each cardiac structure is morphologically recognizable (8–10). The atrial septum has a right and left side with the foramen ovale flap on the left side and the Eustachian valve on the right side. The left and right atrium (RA) can be distinguished by looking at their atrial appendages: the left atrial appendage is thin and long, and the right atrial appendage is wide and triangular. The atrioventricular valves always belong to the appropriate underlying ventricle. The tricuspid valve is composed of three leaflets, lies more toward the apex, has septal attachments and is associated with the morphologic right ventricle. The mitral valve’s two leaflets are attached to the lateral wall of the left ventricle by two papillary muscles with no septal attachment and are associated with the morphologic left ventricle. The right ventricle is heavily trabeculated and has the moderator band while the left ventricle is smoothly walled. The aorta gives rise to the head and neck vessels and the coronary arteries. The pulmonary artery gives rise to the right and left branch pulmonary arteries.

## Standard Windows and Views

**Figure 1:**
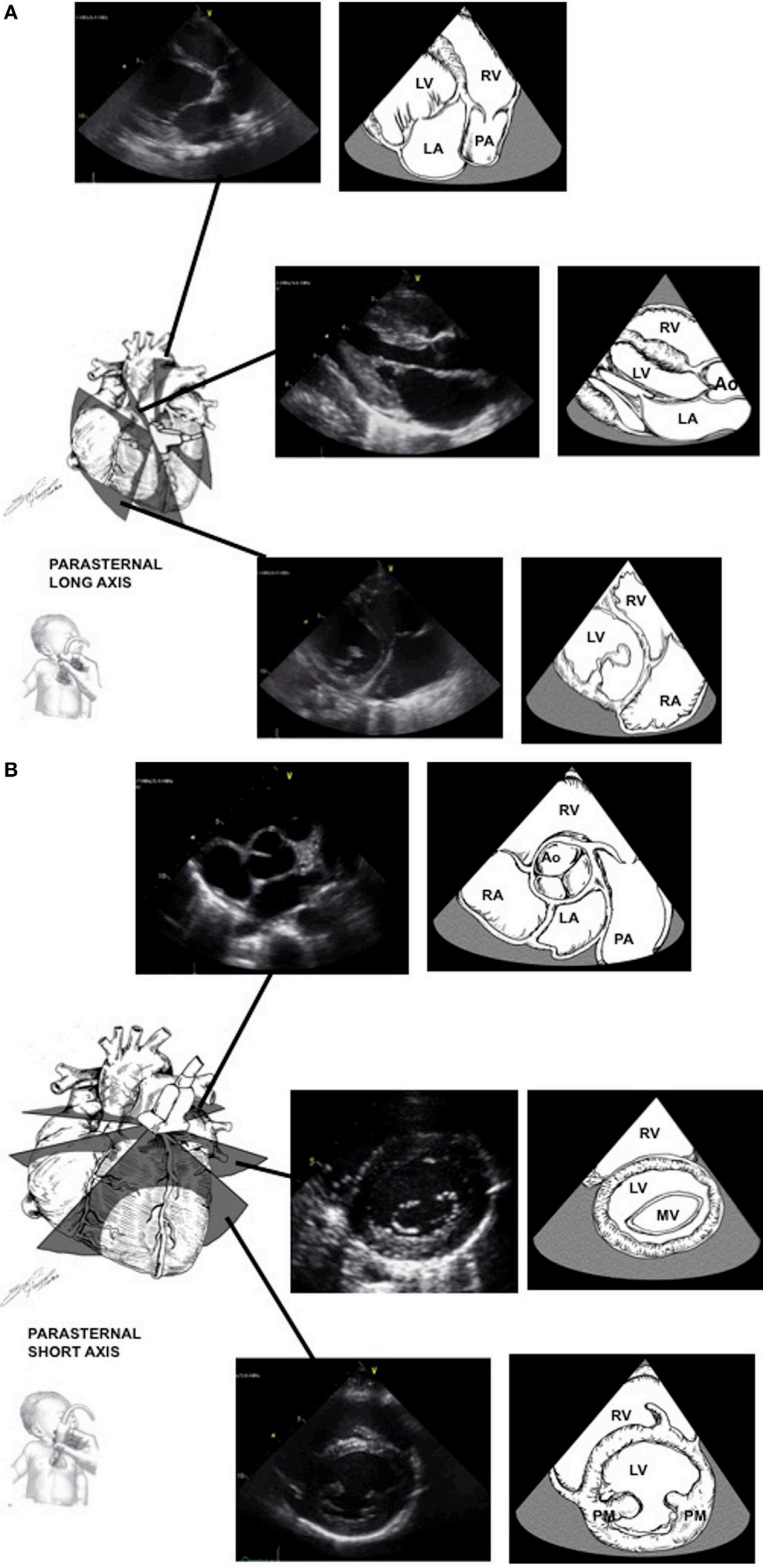

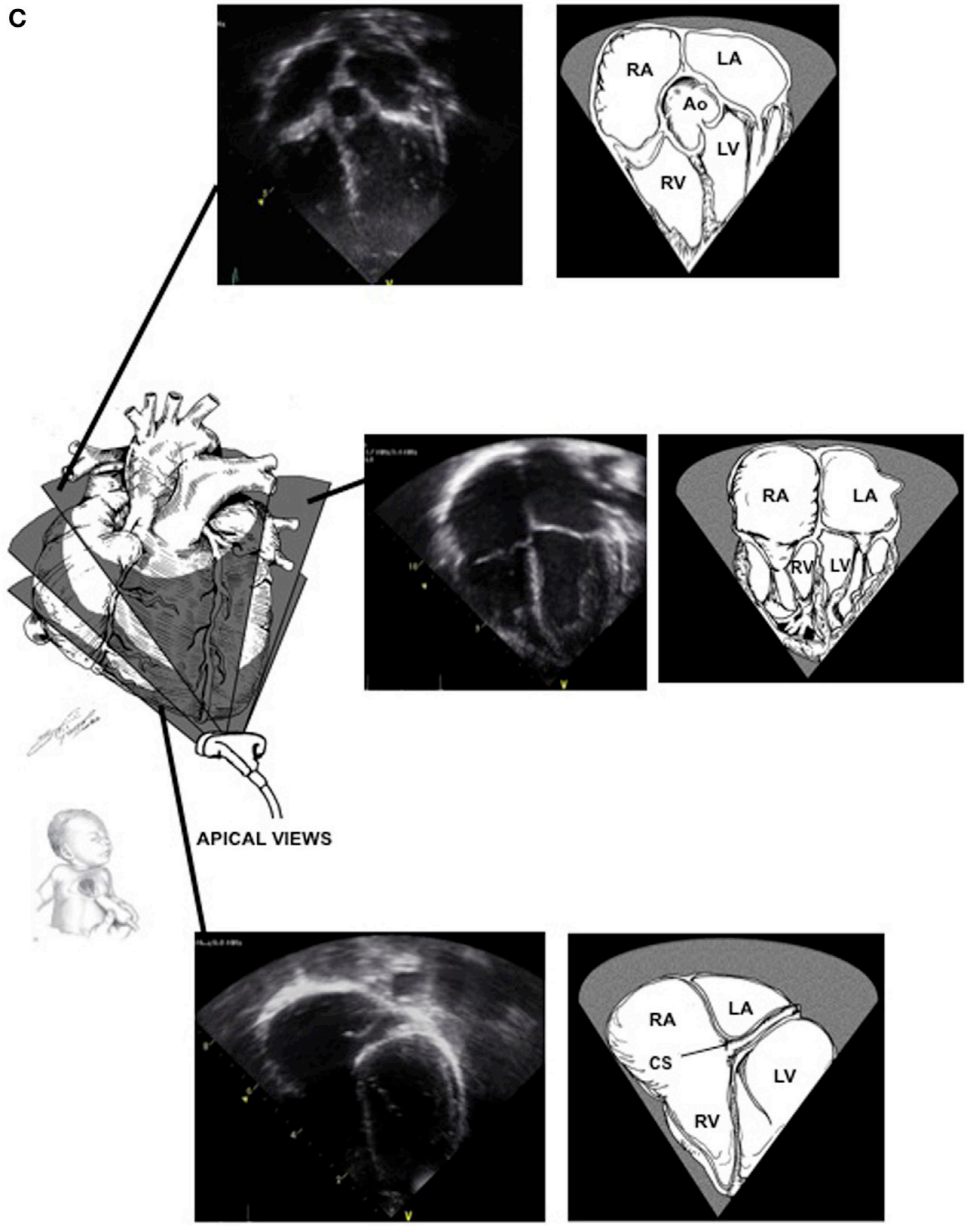

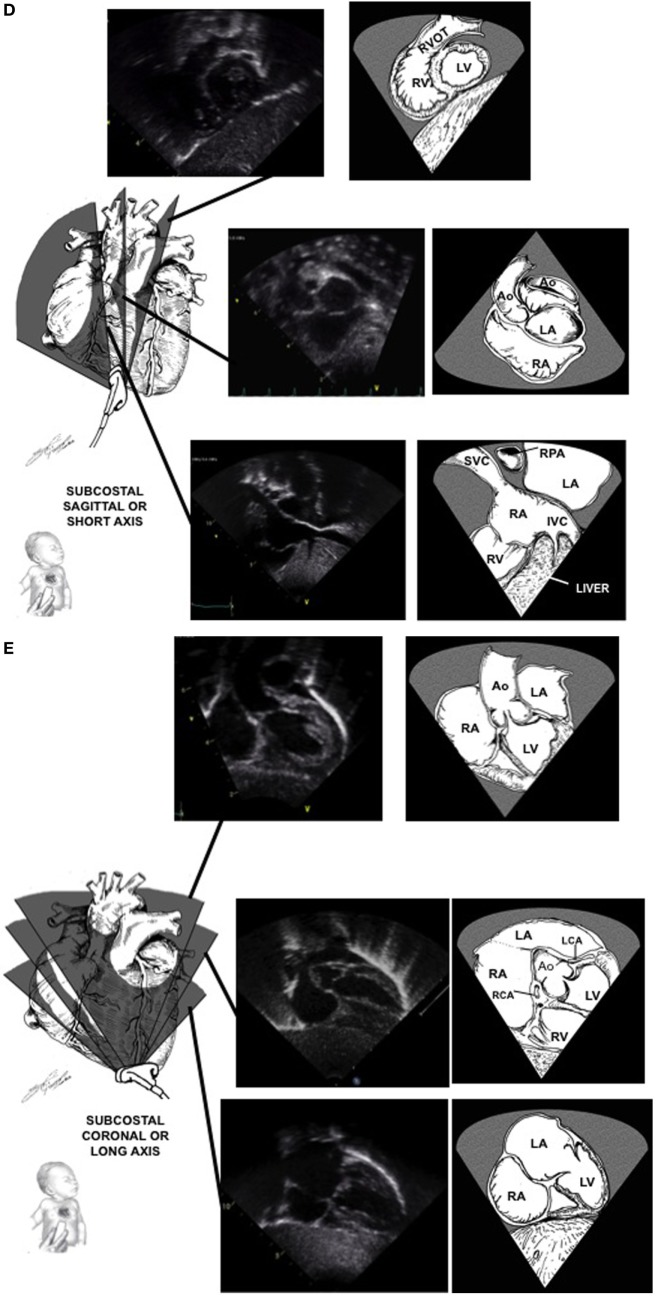

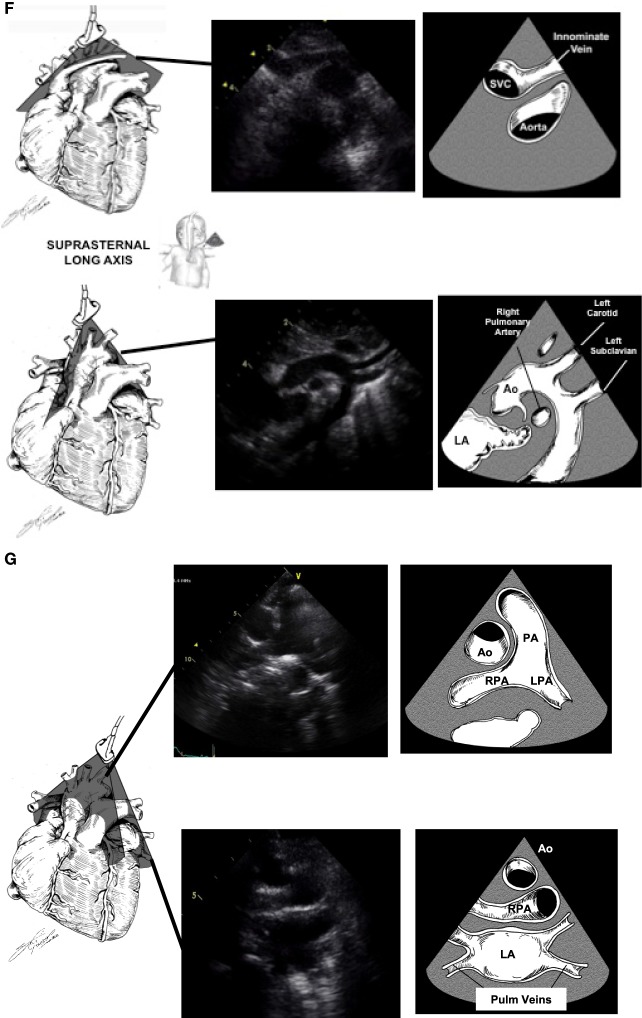
Standard echocardiographic image planes from the high left chest just lateral to the sternum [parasternal window **(A,B)**], the left lateral chest just inferior and lateral to the nipple [apical window **(C)**], sub-xyphoid area [subcostal window **(D,E)**], and the suprasternal notch [suprasternal window **(F,G)**]. Ao, aortic valve; CS, coronary sinus; LA, left atrium; LV, left ventricle; PA, pulmonary artery; RA, right atrium; RV, right ventricle; RVOT, right ventricular outflow tract; SVC, superior vena cava. Adapted from Ref. (1).

The standard windows for echocardiography in children include the parasternal views (high left thorax just lateral to the sternum), the apical views (left lateral thorax just inferior and lateral to the nipple), the subcostal views (below the xiphoid region), and the suprasternal view (in the suprasternal notch) (Figures 1A–F).

### Parasternal Window

The parasternal view looks at the heart aligned along its long and short axis. In the long axis view (Figure 1A), the left ventricular inflow and outflow tracts can be imaged. The aorta, including the annulus, the sinuses of Valsalva, and the proximal portion of the ascending aorta can be observed, as well as its relationship to the mitral valve (mitro-aortic continuity). The left ventricular infero-posterior wall and interventricular septum are visualized. The anterior and posterior leaflets of the mitral valve can be examined. By angulating the transducer posteriorly, the right ventricular inflow tract with the tricuspid valve can be imaged. If the transducer is angulated anteriorly, the right ventricular outflow tract is visualized, including the pulmonary valve. Ninety degrees clockwise rotation will provide a short axis view of the heart (Figure 1B). Short axis views allows for evaluation of the heart chambers, the semilunar and atrioventricular valves, and the coronary arteries. The ventricular chambers can be examined by sweeping from the apex toward the base of the heart. In this view, the left ventricle has a circular shape with symmetric contraction. The right ventricle is trabeculated and crescent-wrapping around the left ventricle. Sweeping further to the base of the heart will show the mitral valve and the papillary muscles. Continuing to the base of the heart will allow imaging of the tri-leaflet aortic valve in the center of the image with the right ventricular outflow tract and pulmonary artery wrapping anteriorly and to the left. Part of the atrial septum and the tricuspid valve may be seen on the right side. Progressive sweep permits the examination of the atrial appendages, ascending aorta in cross-section and branch pulmonary arteries.

### Apical Window

The apical view (Figure 1C) allow for visualization of all four chambers with the heart valves in a left-to-right orientation. The four-chamber view identifies the anatomic right and left ventricles. Sweeping posteriorly will allow visualization of the coronary sinus in the left atrioventricular groove. Sweeping anteriorly will give the five-chamber view, where the atrial and ventricular septa may be imaged and the left ventricular outflow tract and ascending aorta may be examined. The mitral valve leaflets can also be seen in this view, as well as the pulmonary veins as they enter the left atrium. Turning the transducer 60° clockwise will bring a three-chamber view which shows the sub-aortic structures. This represents the best view to assess for Doppler measurement of the left ventricular outflow tract. Turning the transducer 90° counterclockwise from the four-chamber view will produce a two-chamber view of the left ventricle and left atrium. This view is the best to evaluate anterior and posterior left ventricular wall function.

### Subcostal Window

The subcostal view (Figures 1D,E) provides the most comprehensive information. Children are placed supine with the transducer in the subxiphoid position. In older cooperative children, better image quality may be achieved by having the child hold his breath allowing the heart to move downwards toward the transducer. Transverse views should determine visceral situs as well as the relationship of the inferior vena cava and aorta. Sweeps will provide detailed imaging of the atrial and ventricular septum, the atrioventricular valves, the atrial and ventricular chambers, and systemic venous return. Rotating the transducer will permit to image both right and left ventricular outflow tracts. Even the peripheral pulmonary arteries and entire aorta may be examined from this position in some patients. In some children with lung disease or in intensive care patients with poor acoustic windows, this is the best view to obtain adequate imaging of the heart.

### Suprasternal Window

The suprasternal view is obtained by placing the transducer in the suprasternal notch with the child’s neck extended and slightly turned to the left. The suprasternal long and short axis (Figures 1F,G) views give information regarding the side of the aortic arch, the ascending and descending aorta with the head and neck vessels, the size and branching of the pulmonary arteries, as well as anomalies of the systemic and pulmonary venous return. A patent ductus arteriosus can also be imaged in this view.

## M-Mode Echocardiography

**Figure 2:**
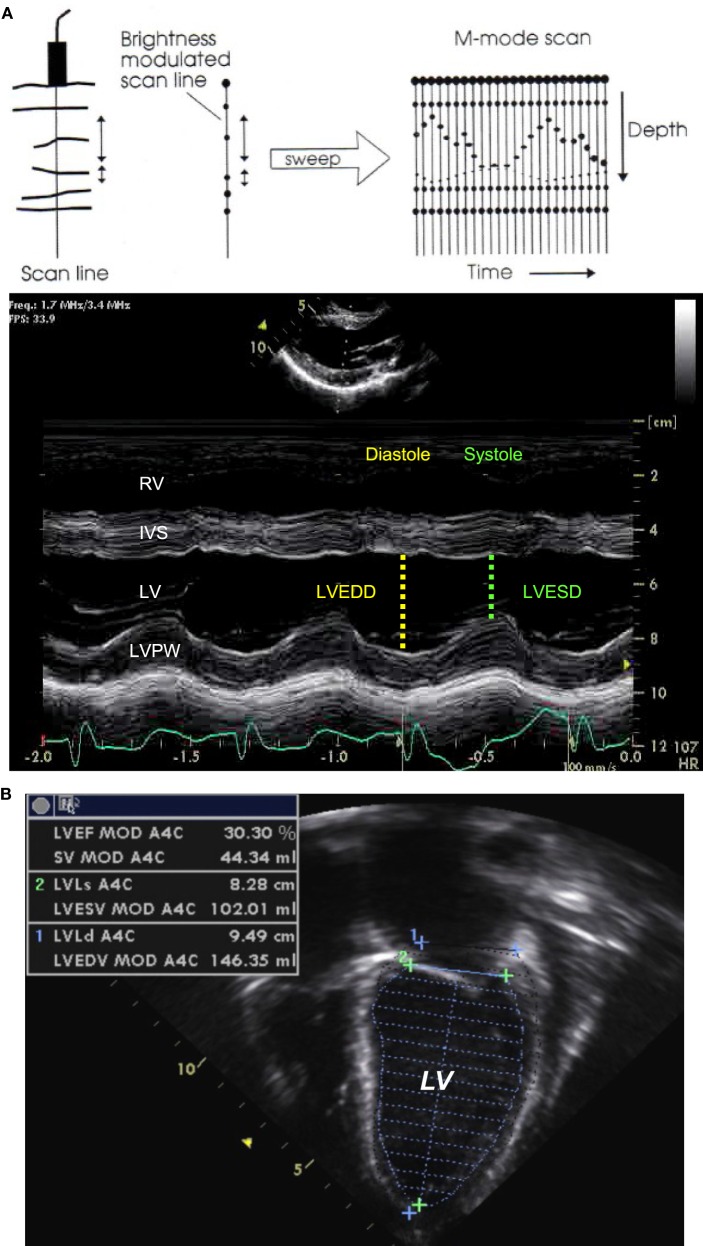
M-mode echocardiography **(A)** obtained from the parasternal long axis view through the right and left ventricular chambers at the tip of the mitral valve leaflets, allowing for the estimation of the fractional shortening. Two-dimensional echocardiography **(B)** obtained from an apical four-chamber view with tracing of the endocardial LV border during end-diastole and end-systole, allowing for estimation of the ejection fraction. IVS, interventricular septum; LV, left ventricle; LVEDD, left ventricular end-diastolic dimension; LVESD, left ventricular end-systolic dimension; LVPW, left ventricle posterior wall; RV, right ventricle. Adapted from Ref. (12).

M-mode echocardiography is useful to evaluate cardiac dimensions, timing with the cardiac cycle and function, and is best obtained from the parasternal long or short axis view (11). The M-mode image is produced by a single line of interrogation that is repeatedly produced, with the picture displayed with the time along the *x* axis and with the distance from the transducer along the *y* axis (Figure 2A). M-mode is mainly used to estimate ventricular function and wall thickness. Left ventricular function can be estimated by measuring the left ventricular dimension in end-diastole and end-systole (LVEDD and LVESD). The fractional shortening (FS) is calculated using the following equation:

$$\text{FS}\left(\text{\%}\right)=\frac{\text{LVEDD}-\text{LVESD}}{\text{LVEDD}}\times \text{100.}$$

This method relies on the assumption of a cylinder shape of the ventricle (Simpson rule), and the estimation is altered in certain circumstances (single ventricle, right ventricle, globular left ventricle).

## 2D Echocardiography

Two-dimensional echocardiography provides direct visual assessment of the beating heart, allowing for evaluation of the size of the cardiac chambers, thickness of the walls, valve and ventricular function, volume status, and presence of pericardial effusion.

The systolic cardiac function depends upon left ventricular contractility, preload, afterload, and heart rate. The cardiac function can be visually assessed and classified into normal or reduced to a mild, moderate, or severe degree. The left ventricular ejection fraction is the best indice of left ventricular function and can be estimated by measuring the left ventricular volume in end-diastole (LVEDV) and end-systole (LVESV) from an apical four-chamber view (Figure 2B). The ejection fraction (EF) is calculated using the following equation:

$$\text{EF}\left(\text{\%}\right)=\frac{\text{LVEDV}-\text{LVESV}}{\text{LVEDV}}\times \text{100.}$$

**Figure 3:**
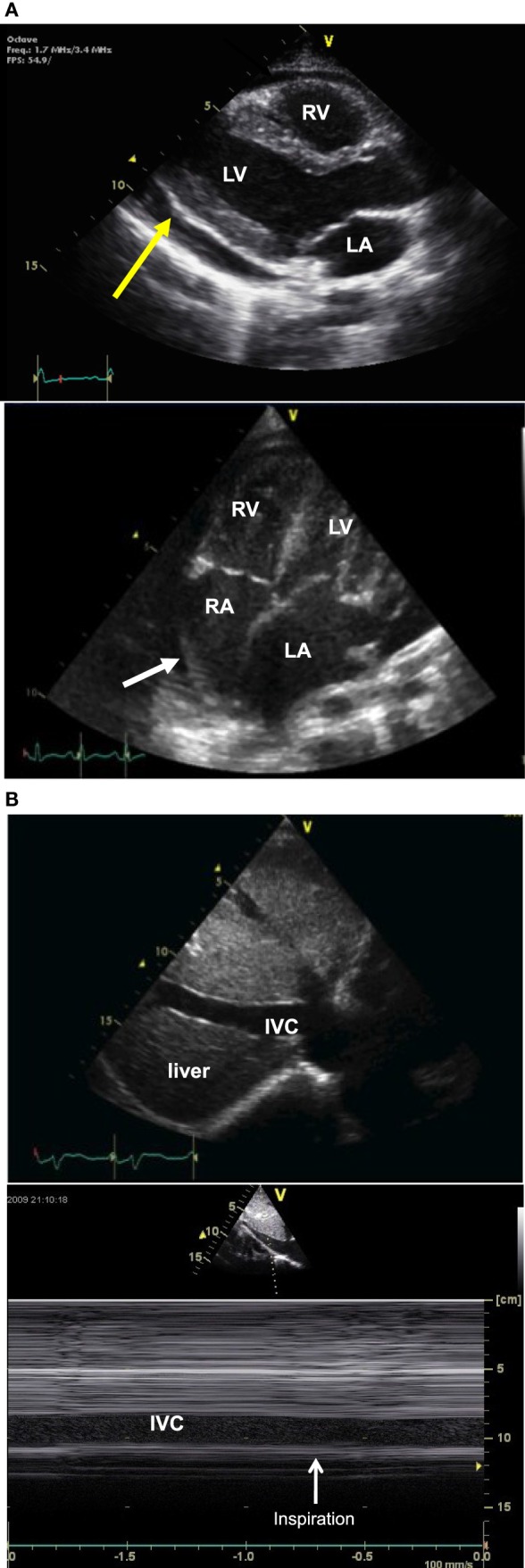
Two-dimensional (2D) echocardiography picture **(A)** obtained from a parasternal long axis view (picture above) showing an echo-free space (yellow arrow) between the two pericardial layer typical of pericardial effusion, and picture obtained from an apical four-chamber view (picture below) showing right atrial free wall collapse (white arrow) consistent with cardiac tamponade. 2D echo picture [**(B)**, above] obtained from a longitudinal subcostal view showing a dilated IVC and M-mode echocardiography [**(B)** below] showing no respiratory variation of IVC size. IVC, inferior vena cava; LA, left atrium; LV, left ventricle; RA, right atrium; RV, right ventricle.

Two-dimensional echocardiography is the method of choice to diagnose pericardial effusion and to assess for cardiac tamponade. Pericardial effusion appears as an echo-free space between the two pericardial layers (Figure 3A), best seen from the parasternal long axis view or from the subcostal views. The size of the echo-free space depends on the amount of fluid and varies with the cardiac cycle. It should always be measured during diastole, to allow for reproducibility. Hemodynamic significance of a pericardial effusion does not depend only on the amount of pericardial fluid and cardiac tamponade can be seen even with small effusion when the accumulation is rapid or with localized effusion as seen after cardiac surgery. Echocardiographic signs of cardiac tamponade are late diastolic right atrial free wall collapse, early diastolic right ventricular collapse, and significant respiratory variation in tricuspid, mitral, and/or aortic (>25%) Doppler flow pattern. Cardiac tamponade is also characterized by increased right heart filling pressure. Echocardiography does not produce accurate estimates of filling pressure but signs of increased right heart filling pressure are a dilated inferior vena cava with loss of respiratory variation (Figure 3B).

## Doppler Echocardiography

Movement of the blood or myocardium is provided by looking for Doppler shift in the reflected ultrasound waves. The Doppler principle is based on the theory that for an immobile object, the frequency of ultrasound reflected is equal to the transmitted frequency. Moving objects change the frequency of the Doppler shift according to the direction and velocity with which they are moving in relation to the transducer (13).

### Spectral Doppler

Intracardiac and vascular hemodynamics may be obtained when velocities are measured. Pulsed-wave (PW), continuous-wave (CW) modes, and color flow Doppler are essential for a complete evaluation in children. Spectral Doppler velocities are recorded for all the valves (mitral, tricuspid, aortic, and pulmonary), the main pulmonary artery and its branches, the ascending and descending aorta, the pulmonary veins, and vena cava. Physiologic tricuspid and pulmonary valve regurgitation is seen in 83 and 93% of normal individuals (14).

Doppler data are typically displayed as velocity. The velocities can then be transformed into pressure data using the modified Bernoulli equation: *P*_1_ *− P*_2_ = 4 [(*V*_2_)^2^ − (*V*_1_)^2^]. Assuming that the level of obstruction and therefore the velocity of V1 is trivial compared with the obstruction at *V*_2_, the formula becomes simpler and is known as the modified Bernoulli equation: Δ*P* = 4(*V*_max_)^2^.

**Figure 4:**
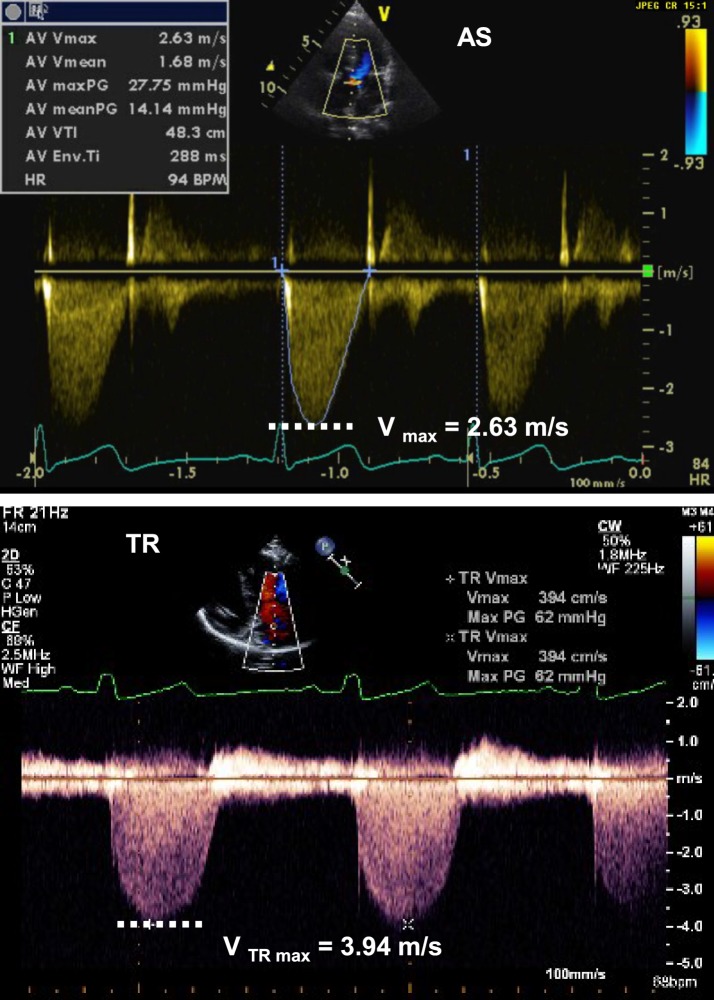
Spectral Doppler tracing (picture above) from aortic valve stenosis (AS) showing *V*_max_ of 2.63 m/s, allowing for estimation of a pressure gradient across the valve of 28 mmHg, consistent with moderate aortic valve stenosis. Spectral Doppler tracing (below picture) from tricuspid regurgitation (TR) showing Vmax of 3.94 m/s, allowing for estimation of a SPAP of 62 mmHg + RAP. RAP, right atrial pressure; SPAP, systolic pulmonary artery pressure.

The modified Bernoulli equation helps estimate the pressure gradient and the severity of stenosis across a valve (Figure 4): When tricuspid regurgitation (TR) is present, the pressure drop across the tricuspid valve during systole reflects the pressure difference between the right ventricle (RV) and right atrium (RA):

$$P_{\text{RV}}-P_{\text{RA}}=4{\left(V_{\text{TR max}}\right)}^{2}\to P_{\text{RV}}=4{\left(V_{\text{TR max}}\right)}^{2}+P_{\text{RA}}.$$

If there is no pulmonary stenosis, the pulmonary artery pressure (PAP) is equal to the right ventricular (RV) pressure during systole. This leads to the estimation of the systolic PAP from the TR jet (Figure 4):

$$\text{Systolic PAP}=\text{[4 (}V_{\text{TR}}{\;}_{\text{max}}{\text{)}}^{2}\text{]}+P_{\text{RA}}\text{.}$$

**Figure 5:**
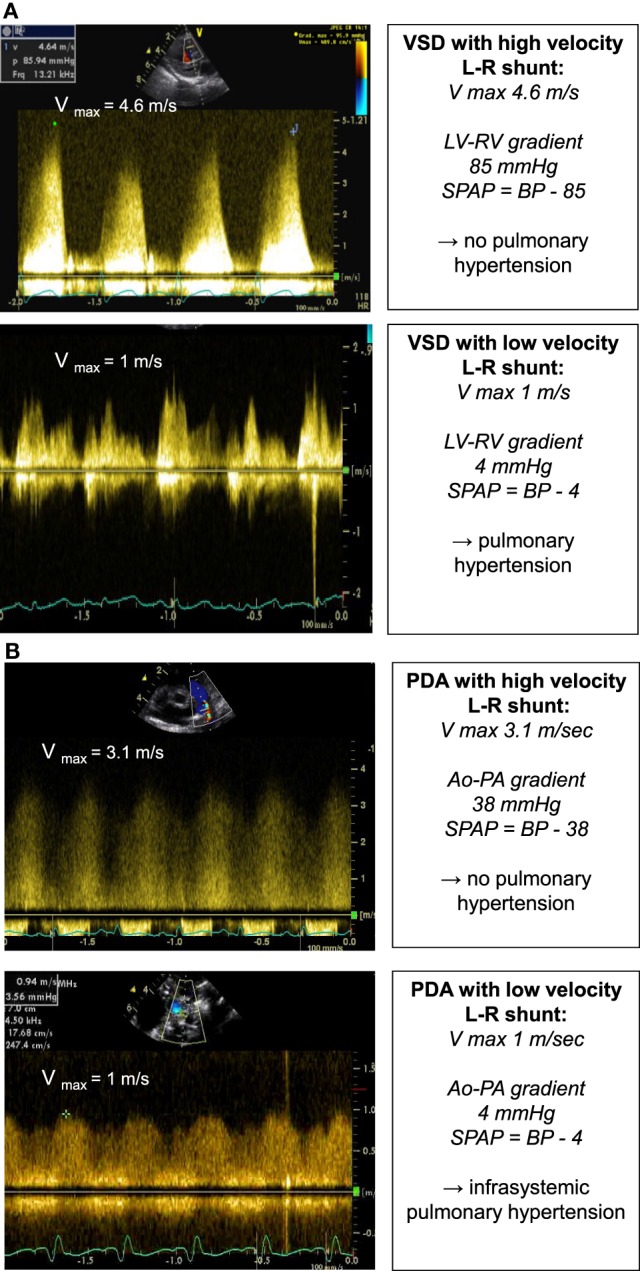
Spectral Doppler **(A)** obtained through a VSD with high velocity left-to-right shunt and no pulmonary hypertension (above picture) and with low velocity left-to-right shunt and pulmonary hypertension (below picture). The pressure gradient across the VSD allows for estimation of the systolic PAP. Spectral Doppler **(B)** obtained through a PDA with high velocity left-to-right shunt and no pulmonary hypertension (above picture) and with low velocity left-to-right shunt and pulmonary hypertension (below picture). The pressure gradient across the PDA allows for estimation of the systolic PAP. BP, systolic blood pressure; LV, left ventricle; PAP, pulmonary artery pressure; PDA, patent ductus arteriosus; RV, right ventricle; VSD, ventricular septal defect.

The same principle can be applied in presence of a ventricular septal defect or patent ductus arteriosus (Figures 5A,B). The pressure drop across the defect allows the estimation of the PAP and prediction of the severity of pulmonary hypertension:

$$\text{Systolic PAP}=\text{ BP}-\text{[4 (}V_{\text{max}}{\text{)}}^{2}\text{],}$$

where BP represents the systolic blood pressure.

During Doppler imaging, it is important that the beam should be perfectly aligned with the line of flow to avoid distortion of data. An angle of interrogation of less than 20° is essential to ensure clinically accurate information.

### Pulse-Wave (PW) and Continuous-Wave (CW) Doppler

**Figure 6:**
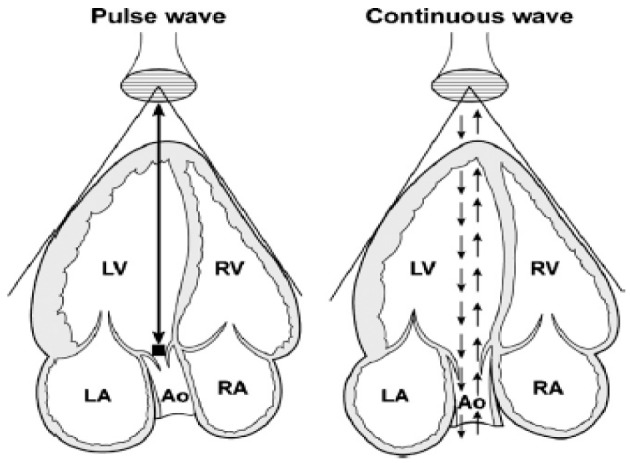
Schematic representation of pulse-wave and continuous-wave Doppler at the level of the aorta from an apical five-chamber view. Ao, aorta; LA, left atrium; LV, left ventricle; RA, right atrium; RV, right ventricle. Adapted from Ref. (16) (Figure 2).

The two used modes for spectral Doppler interrogation are pulsed-wave (PW) and continuous-wave (CW) Doppler. PW Doppler’s main advantage is that it permits sampling the velocity of blood flow at a precise point within the cardiac field (Figure 6). However, the main disadvantage comes from sampling theory, which states that the maximum detectable frequency shift (the Nyquist limit) is equal to half the sampling rate [the pulse repetition frequency (PRF)]. PW is then limited by the Nyquist limit making it unsuitable for high velocity flow quantification. In CW Doppler, the sampling rate in infinite and is not constrained by velocity limits. CW Doppler can record velocities exceeding those of PW imaging but interrogates all points along a given beam. The disadvantage is that knowledge of anatomy must already be determined to identify the precise location of the maximum velocity. Another way to increase the maximum detectable velocity is to use high pulse repetition frequency (HPRF) imaging (15). In HPRF, the pulse repetition rate is set so high that the signal from one pulse does not finish its return before the next pulse is conveyed. Thus, Doppler shift are detected from more than one interrogation site.

**Figure 7:**
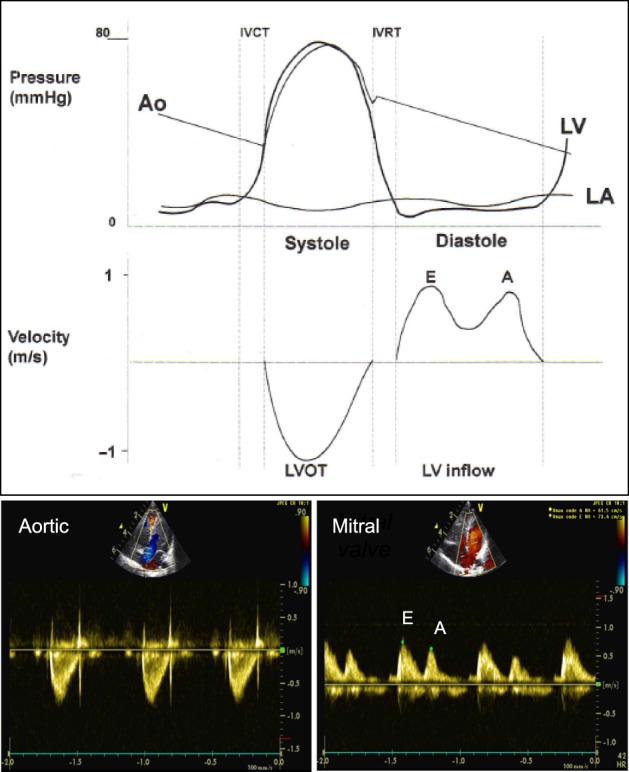
Comparison of pressure curve of the aorta (Ao), left ventricle (LV), left atrium (LA) and velocity across the left ventricular outflow tract (LVOT) and left ventricular inflow (LV inflow) and spectral Doppler from pulse wave across the LVOT (aortic valve) from apical five-chamber view and LV inflow (mitral valve) from apical four-chamber view. E, early diastolic ventricular filling wave; A, late diastolic ventricular filling during atrial contraction wave. Adapted from Ref. (12).

PW Doppler uses shot bursts of ultrasound signals transmitted at regular intervals (PRF). PW is used to record the velocity across the valves, by placing the sample volume slightly proximal to the valve. The pulmonary and aortic valve Doppler tracing is a unique envelope with a peak velocity of approximately 1 m/s (Figure 7). The tricuspid and mitral valve Doppler tracing show two phases: flow in early diastole, representing early passive ventricular filling, is characterized by a peak wave called the E wave. It is followed by the A wave, representing late ventricular filling during atrial contraction (Figure 7) (17). The mitral valve peak velocity is slightly higher than that of the tricuspid valve (18). The flow in the pulmonary vein is continuous with a diastolic (D wave) and a systolic (S wave) peak. The diastolic peak velocity is usually higher than the systolic. Often, flow reversal (A wave) can be seen during atrial contraction. This flow pattern does not vary with respiration (19). Vena cava flow is a continuous low velocity flow but the peak velocity is greater in systole (S wave) compared to diastole (D wave). Flow reversal during atrial contraction (A wave) occurs less often in children compared to adults. Respiratory variation is seen with augmentation of flow velocities during inspiration (17). Hepatic venous flow shows a predominantly systolic biphasic flow pattern (20).

### Color Doppler

**Figure 8:**
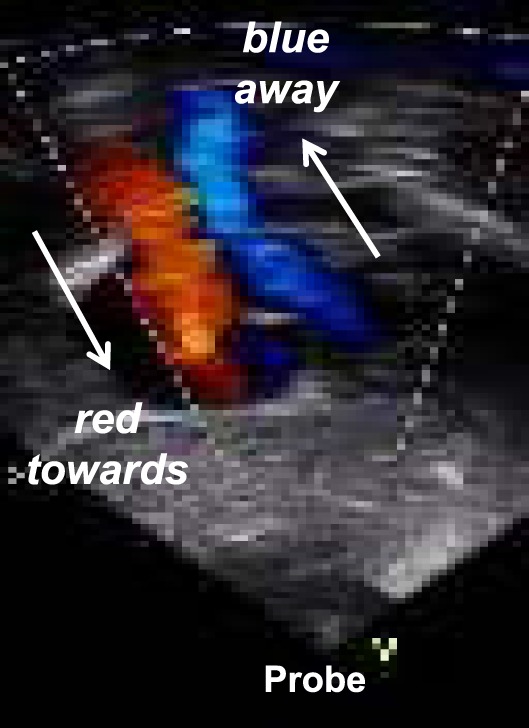
Color Doppler flow mapping with Blue directed away from the probe and red directed toward the probe: mnemotechnic = BART.

Color flow Doppler mapping allows velocity information to be overlaid on a 2D image providing data about intracardiac and extracardiac shunts, regurgitation or stenosis of valves, and vessel obstruction. By convention, shades of red are utilized in identifying blood flowing toward the transducer and blue to indicate blood flowing away from the transducer (Figure 8). Therefore, color flow Doppler defines the presence and direction of blood flow and shunts and is used to grade the severity of valvar regurgitation.

Aliasing occurs with color Doppler or pulse-wave spectral Doppler. With high velocity blood flow causing Doppler shifts above the Nyquist limit, aliasing occurs and is displayed as bright, turbulent color Doppler flow and in blood flow profiles wrapping around the displayed scale in PW spectral Doppler. The tricks to decrease or eliminate aliasing of flow are to image at a shallower depth, increase the insonification angle, use low frequency transducer, use CW Doppler, change the color Doppler scale, or move the display baseline.

### Tissue Doppler

**Figure 9:**
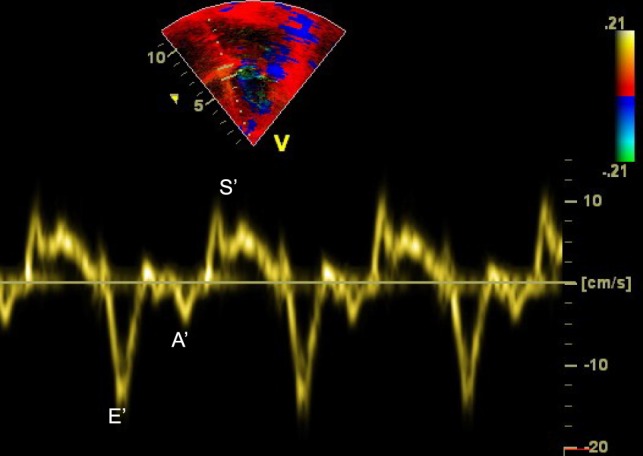
Tissue Doppler imaging (TDI) at the level of the interventricular septum from apical four-chamber view demonstrating the *E*′, *A*′, and *S*′ waves. *A*′, late diastolic ventricular filling during atrial contraction wave; *E*′, early ventricular filling wave; *S*′, systolic wave.

Doppler can be applied to measure myocardial rather than blood velocities. The difference resides in filtering: imaging myocardial velocities requires filtering structures that are moving at high velocity with low scattering power (i.e., the blood) while imaging blood velocity requires filtering out slowly moving and strongly reflecting structures (i.e., the myocardium). Tissue Doppler imaging is helpful for assessing systolic and diastolic myocardial function (21–24). In a four-chamber view, the cursor should be placed at the junction between the ventricles and atria over the interventricular septum, on the lateral wall of the left ventricle and on the lateral wall of the right ventricle. The tracing includes early diastolic (*E*′), late diastolic (*A*′), and systolic (*S*′) waves (Figure 9). Age based normal values in children are available (25–27).

## Current Clinical Applications of Functional Echocardiography in the Pediatric and Neonatal Intensive Care Unit

Clinical applications of echocardiography within the pediatric and neonatal intensive care unit may be divided into the following topics, covered in detail in separate articles published in Frontiers into the research topic TINEC:

– Evaluation of cardiac function;
– Evaluation of filling pressure and fluid responsiveness;
– Evaluation of hemodynamics;
– Diagnosis of pulmonary hypertension;
– Diagnosis of pericardial effusion and tamponade;
– Evaluation of transitional circulation of the newborn;
– Evaluation of ductus arteriosus;
– Evaluation of line placement;
– Guidance of interventions within the intensive care unit.

## Conclusion

Functional echocardiography is a bedside tool able to provide physiological information that may have a significant impact on the management of patients in the intensive care unit. It is useful for real-time assessment of hemodynamics and monitoring of therapeutic interventions. Appropriate training in echocardiography should be incorporated into the intensive care curriculum.

## Author’s Note

CT is a pediatric cardiologist trained at the University Hospital of Geneva, Switzerland, and in Denver, Colorado, USA. CT has been working as an attending pediatric cardiologist at the Children’s Hospital of Geneva (HUG) until recently and is now working in the Pediatric Center at Clinique des Grangettes, Switzerland. NS is a pediatric cardiologist trained at the University Hospital of Lausanne and in Washington University, Saint-Louis, USA. NS is working as the chief of the Pediatric Cardiology Unit at the Centre Hospitalier Universitaire Vaudois (CHUV). VM is a neonatologist trained at the University Hospital of Lausanne, Switzerland, in Denver, Colorado, USA, and in Sydney, Australia. VM has been working as an attending neonatologist at the CHUV until recently and is now working in the Pediatric Service at Hopital du Jura, Delemont, Switzerland. The three authors are part of the organizing committee of the Training in Intensive Care and Neonatal Echocardiography (TINEC), a course on point-of-care echocardiography that is taking place in Lausanne, Switzerland, since January 2016.

## Author Contributions

All authors listed have made a substantial, direct and intellectual contribution to the work, and approved it for publication.

## Conflict of Interest Statement

The authors declare that the research was conducted in the absence of any commercial or financial relationships that could be construed as a potential conflict of interest.
